# The role of self-control and cognitive functioning in educational inequalities in adolescent smoking and binge drinking

**DOI:** 10.1186/s12889-017-4753-2

**Published:** 2017-09-16

**Authors:** Lisa E. M. Davies, Mirte A. G. Kuipers, Marianne Junger, Anton E. Kunst

**Affiliations:** 1 Department of Public Health, Academic Medical Center – University of Amsterdam, P.O. Box 22660, 1100 DD Amsterdam, The Netherlands; 2 Department Industrial Engineering and Business Information Systems (IEBIS), University of Twente, P.O. Box 217, 7500 AE Enschede, The Netherlands

## Abstract

**Background:**

Large differences in substance use between educational levels originate at a young age, but there is limited evidence explaining these inequalities. The aim of this study was to test whether a) smoking and binge drinking are associated with lower levels of self-control and cognitive functioning, and b) associations between educational track and smoking and binge drinking, respectively, are attenuated after controlling for self-control and cognitive functioning.

**Methods:**

This study used cross-sectional survey data of 15 to 20-year-olds (*N* = 191) from low, middle, and high educational tracks. We measured regular binge drinking and regular smoking (more than once a month), cognitive functioning (cognitive ability, reaction time and memory span), and self-control. Logistic regression models were used to assess the associations between educational track and smoking and binge drinking controlled for age, gender and social disadvantage, and for self-control and cognitive functioning.

**Results:**

According to models that controlled for age, gender and social disadvantage only, respondents in the low educational track were more likely to drink heavily (OR = 3.25, 95% CI = 1.48–7.17) and smoke (OR = 5.74, 95% CI = 2.31–14.29) than adolescents in the high educational track. The association between educational track and binge drinking was hardly reduced after adjustment for self-control and cognitive ability (OR = 2.88, 95% CI = 1.09–7.62). Adjustment for self-control and cognitive functioning, especially cognitive ability, weakened the association between education and smoking (OR = 3.40, 95% CI = 1.11–10.37). However, inequalities in smoking remained significant and substantial.

**Conclusions:**

In this study population, pre-existing variations between adolescents in terms of self-control and cognitive functioning played a minor role in educational inequalities in smoking, but not in binge drinking.

## Background

Alcohol consumption and tobacco smoking are amongst the most important causes of mortality and disease burden worldwide causing, respectively, 2.3 and 6.4 million annual deaths and the loss of 85 and 168 million disability-adjusted life years [1]. Both alcohol and tobacco use find their origins in adolescence [2, 3]. In Europe, 35% of 15–16 year old adolescents reported binge drinking (having had five drinks or more on one occasion in the last 30 days) and 22% of reported smoking in the last 30 days [4].

More highly educated individuals enjoy better health and have longer life expectancies than less educated people [5]. Smoking is consistently more prevalent in lower educated adolescents [6–8]. Higher binge drinking rates have also been found in adolescents enrolled in lower educational tracks [7]. Inequalities according to other indicators of socioeconomic status, including parental education, are however less consistent [9, 10]. Understanding the underlying causes of these educational differences in smoking and binge drinking is important to inform strategies aimed at reducing health inequalities already at an early age.

Several factors may contribute to socioeconomic inequalities in adolescent smoking and binge drinking [11–15]. Some quantitative studies have attempted to empirically assess the contribution of these factors. The focus of most of these studies was on the role of social and environmental influences, such as parenting practices [7, 16], parental smoking [17], and peer influences [7, 18–20]. However, educational differences were not fully explained by these factors, suggesting a role of mechanisms not involving these socio-environmental factors.

Pre-existing factors, i.e. factors that are already largely shaped before adolescence, are rarely taken into account when investigating educational differences in health behaviours in adolescence. These pre-existing factors may drive “indirect selection” mechanisms by affecting both how adolescents are being sorted into lower education levels and by directly affecting the likelihood of uptake of smoking and drinking [15]. Two important pre-existing factors include self-control and cognitive functioning. Self-control is an umbrella construct that bridges concepts such as impulsivity, conscientiousness, self-regulation, and willpower [21]. It is regarded the capacity to change, adapt and override one’s response and thus to refrain from high-risk behaviour [21]. Cognitive functioning is a concept that captures various capacities of the brain such as: memory, perception, ability of abstract reasoning, attention span and concentration, problem solving and judgement, and learning ability [22].

Several studies showed that low self-control was associated with increased likelihood of smoking and binge drinking in adolescence [23–25]. Adolescents with higher cognitive ability are less likely to smoke [26], but the association with binge drinking has to our knowledge not been investigated. Self-control and cognitive functioning are also associated with academic achievement [21, 27, 28]. For example, a study by Colom et al. found that basic cognitive functioning (e.g. fluid intelligence and working memory) and temperament difficulties (e.g. sensation seeking and impulsiveness, which both are related to self-control) predict 60% of the variance in academic performance [27]. If adolescents with poorer self-control and cognitive functioning are more likely to initiate substance use as well as to perform poorer in school, this mechanism may contribute to educational inequalities in adolescent substance use.

This study aimed to test whether a) smoking and binge drinking are associated with lower levels of self-control and cognitive functioning, and b) associations between educational track and smoking and binge drinking, respectively, are attenuated after controlling for self-control and cognitive functioning. With this study we provide the first evidence from the Netherlands regarding the role of self-control and cognitive functioning in the association between adolescents’ educational track and smoking and binge drinking behaviour, respectively.

## Methods

### Study population

The current study used the data as described in Junger and Van Kampen [29]. The study was based on a convenience sample consisting of 201 adolescents between 15 and 20 years of age. Ten individuals were excluded due to missing values, which resulted in a study population of 191. Surveys and cognitive tests were performed in 2008, during school hours. Participants were enrolled in eight different schools, located in cities in the centre and south of the Netherlands (Eindhoven, Sleeuwijk, Culemborg, Arnhem, Utrecht, Huizen, and Oosterhout). Schools provided between 8 and 52 respondents (25 on average).

The cognitive tests were performed in either a separate room or a quiet area with three to six students at a time, and were supervised by at least one researcher. Participants first performed the reaction times test, followed by the Raven’s Standard Progressive Matrices, the Corsi block-tapping task, the self-control questionnaire, and finally the general survey (see below). More details on the procedure are published elsewhere [29].

Participation by respondents was entirely voluntary and with minimal risks of any kind, and does therefore comply with the Declaration of Helsinki. The questionnaires and simple tests did not entail observation methods that may be intrusive to subjects. This study does therefore not fall under the Dutch ‘law for medical research’ and ethical approval and active parental consent were therefore not required. To guarantee an ethical procedure, we followed four steps. First, permission for the implementation of the study was implied by the ethical committee of the Faculty of Social Science (FSS) of Utrecht University (which is a former employer of the third author). Second, permission for data collection was requested at each school. For this, all schools discussed the study with their School Board, a board that also included parent representatives. Third, schools informed parents about the study and offered parents the opportunity to opt-out if they disagreed with the participation of their child (passive consent). Fourth, before participating, students were asked to sign a consent form for the use of the data, based on an introductory letter and a brief oral description of the purpose of the study.

### Measurements

*Binge drinking* (six or more drinks on one occasion) and *smoking* frequency were defined in the general survey as ‘binge drinking/smoking once a month or less’ or ‘more than once a month’, with the latter coded as one. Sensitivity analyses were performed with the measures ‘ever binge drinking/smoking' (vs. never) and ‘at least weekly smoking’ (vs. less than weekly). The prevalence of weekly binge drinking was too low to analyse it as an outcome.

*Educational track* measures the level of difficulty of the school curriculum and the lengths of the secondary school career. Schools in the Netherlands may combine different tracks in one building, or offer one specific educational track. In our sample, four schools combined all three tracks, three schools provided only the low track, and one school was a high track school. Respondents were divided into three educational tracks: low, midlevel, and high educational track. Low educational track represents (pre-preparatory) vocational secondary education (provides access ISCED level 3–4), midlevel educational track represents intermediate general education (provides access to ISCED level 5–6) and high educational track represents preparatory university education (provides access to ISCED level 6–8) [30]. High educational track was coded as the reference value.

*Self-control* was measured with the Dutch Version of the Self-control Scale [21]. This questionnaire consisted of 36 statements (e.g. “I do a lot of things without thinking”) rated on a 5-point Likert scale, ranging from ‘not at all like me’ to ‘very much like me’. Because the relationship between self-control and substance use was examined, we excluded 6 health-related items (“I do certain things that are bad for me, if they are fun”, “I refuse things that are bad for me”, “I can resist temptations well”, “I participate in healthy activities”, “I eat healthy”, “Sometimes I abuse drugs or alcohol”) in order to prevent that self-control directly measured health behaviour. Negatively formulated items were reverse-scored. Cronbach’s alpha for the 30 remaining items was 0.84 indicating a high internal reliability. A sum score was calculated with a maximum of 150. A higher score indicated a stronger self-control.

*Cognitive functioning* was measured using three variables; cognitive ability, reaction time and memory span [22].

*Cognitive ability* was measured by the Raven Standard Progressive Matrices (RSPM) [31]. This test measures general cognitive ability (also known as Spearman’s g), the ability to solve problems and understand the relationship of various concepts without using language. The RSPM consisted of 60 items in which participants had to select the missing part (6–8 options) of a sequence of figures. Every correct item accounted for one point resulting in a maximum score of 60. Higher scores indicated higher cognitive ability. The RSPM is a valid and reliable measure for cognitive ability [31].

*Reaction time* was measured with the Simple Reaction Time (SRT) test. The test measured the time between a circle turning green and the subject pushing a button in reaction to the change in colour. The SRT test tests attention and motor speed [32]. There were two blocks, both consisting of 24 trials. The reaction time for all 48 trials was summed and the median of this sum score was used as the reaction time in the analysis. A lower score corresponds to a better cognitive functioning.

*Memory span* was measured using the Corsi block tapping test. The test consists of multiple trials in which participants were asked to recall a sequence in forwards and backwards order. The number of items in the sequence was increased with each trial. The longest sequence length that was correctly recalled at least twice was multiplied by the number of correct trials, resulting in two memory span scores (forwards and backwards). The maximum score was 162. A higher score means better memory span. The Corsi test is a valid measure for short term memory and executive control [33].

*Socio-demographic variables* age, gender, and social disadvantage were measured in the general survey. Age was a continuous variable ranging from 15 to 20. Gender was coded 0 for males and 1 for females. Social disadvantage was measured with four items: family income (1 = less than average, 2 = average, 3 = more than average), occupation of the primary wage earning parent (1 = owner of a large business, executive, 2 = owner of a small business, professional, 3 = semi-professional, skilled labourer, 4 = clerical staff, 5 = semi-skilled worker, 6 = labourer or service worker), ownership of the home (1 = rent vs. 2 = own) and living arrangement (1 = a(n) apartment, flat, single family home, 2 = a detached house, 3 = a mobile home). One point was assigned when income was (less than) average, occupation was manual (options 3–6), parents rented the home, and if the adolescents indicated to live in a flat or mobile home. The sum score for social disadvantage therefore had a minimum of 0 points and a maximum of 4 points.

### Statistical analysis

We used logistic regression models to study the associations of standardised self-control and cognitive functioning variables with smoking and binge drinking, respectively. We applied crude and adjusted models; the adjusted models controlled for age, gender, social disadvantage and educational track. Self-control and cognitive functioning variables were standardized, to enable comparison between these variables. Correlations between self-control and cognitive functioning variables were overall weak, ranging from −0.28 to 0.27.

The association between educational track and smoking and binge drinking, respectively, was analysed using four logistic regression models. Model 1 included educational track, age, gender and social disadvantage. In Model 2 self-control was added and in Model 3 cognitive ability was added. Finally, Model 4 included reaction time and memory span (forwards and backwards). The fit of the models was compared using likelihood ratio tests, in order to determine whether the addition of variables improved the model. In a sensitivity analysis, the explanatory variables were added to Model 1 separately. We found that this alternative approach did not change our conclusions with regards to the extent to which the model fit changed for each of these variables.

The regression analyses were repeated for ever binge drinking/smoking and for weekly smoking. Statistical analyses were performed in Stata version 14.2.

## Results

The characteristics of the respondents are presented in Table 1. There was about an equal proportion of boys and girls, and respondents had a mean age of 16.8 years. Forty-two percent of respondents reported binge drinking at least several times a month, and 30 % of respondents reported smoking at least several times a month. Compared with students in the low educational track, students in the higher educational track had higher levels of general intelligence, faster reaction times, better backward short-term visuospatial memory span and showed lower smoking and binge drinking prevalence rates. Social disadvantage scores, forward memory span and self-control were not consistently different between educational tracks.

**Table 1:** Description of the study population, stratified by educational track

	Total population	Educational track		
		Low	Middle	High
N	191	57	59	75
Age (years)^a^	16.8 (16.6–16.9)	16.9 (16.7–17.2)	16.6 (16.4–16.9)	16.8 (16.6–17.0)
Gender				
Male^b^	49.2 (42.1–56.3)	49.1 (36.3–62.1)	45.8 (33.4–58.7)	52.0 (40.6–63.2)
Female^b^	50.8 (43.7–57.9)	50.9 (38.0–63.7)	54.2 (41.3–66.6)	48.0 (36.8–59.4)
Binge drinking				
Once a month or less^b^	58.1 (50.9–65.0)	47.4 (34.7–60.4)	55.9 (43.0–68.1)	68.0 (56.5–77.6)
More than once a month^b^	41.9 (35.0–49.1)	52.6 (39.5–65.3)	44.1 (31.9–57.0)	32.0 (22.4–43.5)
Smoking				
Once a month or less^b^	70.2 (63.2–76.3)	59.6 (46.3–71.7)	59.3 (46.3–71.1)	86.7 (76.8–92.7)
More than once a month^b^	29.8 (23.7–36.8)	40.4 (28.3–53.7)	40.7 (28.8–53.7)	13.3 (7.27–23.2)
Social disadvantage^a^	1.11 (0.94–1.28)	1.46 (1.11–1.80)	1.00 (0.69–1.31)	0.93 (0.69–1.18)
Self-control^a,c^	95.7 (93.8–97.7)	96.7 (93.3–100.1)	89.9 (85.9–92.9)	100.0 (97.0–102.9)
Cognitive functioning				
Cognitive ability (g)^a,c^	46.6 (45.6–47.6)	41.3 (39.5–43.0)	46.5 (45.0–48.0)	50.7 (49.6–51.7)
Reaction time (ms)^a,d^	273.0 (268.5–277.5)	283.5 (274.6–292.5)	279.0 (270.6–287.3)	260.3 (255.0–265.6)
Memory span Forwards^a,c^	49.7 (45.8–53.5)	44.3 (37.5–51.1)	50.7 (44.1–57.2)	52.9 (46.5–59.4)
Memory span Backwards^a,c^	37.7 (34.6–40.8)	29.4 (23.5–35.2)	37.0 (32.0–42.0)	44.5 (39–8-49.2)

^a^Mean (95% CI)

^b^Percentage (95% CI)

^c^higher scores are favourable

^d^lower scores are favourable

Table 2 presents smoking and binge drinking prevalence rates for respondents scoring under and above the median of self-control and cognitive functioning. Smoking and binge drinking were more prevalent in those with less favourable scores on self-control, cognitive ability, reaction time and memory span forwards. However, higher smoking and binge drinking rates were observed in those with higher scores on memory span backwards. These findings are reflected in the odds ratios presented in Table 2. Crude logistic regression analyses show that higher self-control scores were associated with lower odds of binge drinking. Higher scores for cognitive ability and self-control were associated with lower odds of smoking. After controlling for age, gender, social disadvantage and educational track, only the association of self-control with smoking and binge drinking could be demonstrated with statistical significance (OR binge drinking = 0.61, 95% CI = 0.43–0.86; OR smoking = 0.55, 95% CI = 0.37–0.82).

**Table 2:** Prevalence rates of smoking and binge drinking in students with scores under and above the median for self-control and cognitive functioning variables, and the association of standardised self-control and cognitive functioning with smoking and binge drinking

	Prevalence rates with 95% CI		Odds ratios with 95% CI	
	≤ Median	> Median	Crude model	Adjusted model^a^
Binge drinking				
Self-control^b^	54.6 (45.6–64.3)	28.7 (20.4–38.8)	0.58 (0.42–0.79)	0.61 (0.43–0.86)
Cognitive ability^b^	42.7 (33.1–52.9)	41.1 (31.5–51.3)	0.80 (0.60–1.07)	1.02 (0.70–1.49)
Reaction time^c^	51.0 (41.0–61.0)	32.6 (23.9–42.8)	0.89 (0.66–1.19)	0.89 (0.63–1.25)
Memory span Forwards^b^	45.5 (35.8–55.4)	38.0 (28.6–48.5)	0.78 (0.58–1.04)	0.73 (0.52–1.02)
Memory span Backwards^b^	39.2 (29.9–49.3)	44.7 (34.9–54.9)	1.09 (0.81–1.45)	1.23 (0.89–1.70)
Smoking				
Self-control^b^	43.3 (33.7–53.4)	16.0 (9.78–24.9)	0.50 (0.35–0.70)	0.55 (0.37–0.82)
Cognitive ability^b^	36.5 (27.4–46.6)	23.3 (15.6–32.8)	0.60 (0.44–0.83)	0.76 (0.51–1.13)
Reaction time^c^	25.0 (17.3–34.7)	34.7 (25.8–44.9)	1.29 (0.95–1.75)	1.25 (0.87–1.78)
Memory span Forwards^b^	33.3 (24.7–43.3)	26.1 (18.1–36.1)	0.93 (0.68–1.27)	1.01 (0.71–1.44)
Memory span Backwards^b^	24.7 (17.1–34.4)	35.1 (26.1–45.4)	1.02 (0.75–1.39)	1.27 (0.90–1.81)

^a^Controlled for age, gender, social disadvantage, and educational track

^b^higher scores are favourable

^c^lower scores are favourable

Estimates of educational differences in binge drinking are presented in Table 3. Adolescents in the low educational track were more likely to binge drink than those in the high educational track (OR = 3.25, 95% CI = 1.48–7.17). Controlling for self-control (Model 2) reduced the association between educational track and binge drinking (OR = 2.90, 95% CI = 1.30–6.48) and the model fit significantly improved (*p* = 0.004). Controlling for cognitive ability hardly reduced the odds ratio (OR = 2.88, 95% CI = 1.09–7.62), but resulted in a worse model fit. Adding reaction time and memory span did not significantly improve the model and returned the odds ratio back to its original value of 3.25. This was due to the positive association between memory span and binge drinking. Educational differences in binge drinking remained significant in all four models.

**Table 3:** Stepwise controlled models of the association between educational track and binge drinking (more than once a month vs. once a month or less)

	Odds ratio’s and 95% confidence intervals			
	Model 1	Model 2	Model 3	Model 4
Educational track				
High	1.00	1.00	1.00	1.00
Middle	2.03 (0.95–4.36)	1.41 (0.62–3.21)	1.41 (0.59–3.35)	1.50 (0.60–3.72)
Low	3.25 (1.48–7.17)	2.90 (1.30–6.48)	2.88 (1.09–7.62)	3.25 (1.17–9.02)
Gender				
Male	1.00	1.00	1.00	1.00
Female	0.32 (0.17–0.61)	0.33 (0.17–0.63)	0.33 (0.17–0.63)	0.31 (0.16–0.61)
Age	1.18 (0.86–1.63)	1.16 (0.84–1.60)	1.16 (0.84–1.60)	1.11 (0.79–1.56)
Social disadvantage	0.69 (0.52–0.92)	0.72 (0.54–0.96)	0.72 (0.55–0.96)	0.70 (0.52–0.95)
Self-control^a^		0.61 (0.43–0.86)	0.61 (0.43–0.86)	0.59 (0.41–0.84)
Cognitive ability^a^			0.99 (0.68–1.46)	1.04 (0.70–1.56)
Reaction time^b^				0.92 (0.64–1.56)
Memory span Forwards^a^				0.66 (0.46–0.95)
Memory span Backwards^a^				1.20 (0.85–1.70)
Model fit AIC, *p*-value^c^	240.6, <0.001	234.3, 0.004	236.3, 0.979	235.8, 0.092

^a^higher scores are favourable

^b^lower scores are favourable

^c^Model Akaike’s Information Criterion (AIC) and p-value of likelihood ratio test comparing model fit with the less complex nested Model, i.e. Model 1 compared with a crude Model, Model 2 compared with Model 1, Model 3 compared with Model 2, and Model 4 compared with Model 3

Table 4 presents the results for smoking. Adolescents in the low educational track were significantly more likely to smoke than those in the high educational track (OR = 5.74, 95% CI = 2.31–14.29). Controlling for self-control (Model 3) reduced this association to a minor extent (OR = 5.27, 95% CI = 2.09–13.30) and improved model fit. Controlling for cognitive ability (Model 3) reduced the association substantially (OR = 3.42, 95% CI = 1.18–9.92) and improved model fit. Controlling for reaction time and memory span did not change the association (OR = 3.40, 95% CI = 1.11–10.37). Educational differences remained significant and substantial in all four models.

**Table 4:** Stepwise controlled models of the association between educational track and smoking (more than once a month vs. once a month or less)

	Odds ratios and 95% confidence intervals			
	Model 1	Model 2	Model 3	Model 4
Educational track				
High	1.00	1.00	1.00	1.00
Middle	5.92 (2.39–14.68)	4.00 (1.54–10.37)	3.22 (1.20–8.65)	3.02 (1.08–8.42)
Low	5.74 (2.31–14.29)	5.27 (2.09–13.30)	3.42 (1.18–9.92)	3.40 (1.11–10.37)
Gender				
Male	1.00	1.00	1.00	1.00
Female	0.76 (0.38–1.51)	0.81 (0.39–1.65)	0.86 (0.42–1.79)	0.82 (0.39–1.74)
Age	1.64 (1.14–2.35)	1.64 (1.14–2.37)	1.62 (1.12–2.34)	1.74 (1.19–2.55)
Social disadvantage	0.69 (0.50–0.95)	0.72 (0.52–1.00)	0.71 (0.51–1.00)	0.67 (0.47–0.95)
Self-control^a^		0.55 (0.37–0.82)	0.53 (0.36–0.80)	0.52 (0.35–0.79)
Cognitive ability^a^			0.72 (0.57–1.08)	0.70 (0.46–1.08)
Reaction time^b^				1.40 (0.94–2.11)
Memory span Forwards^a^				1.05 (0.71–1.56)
Memory span Backwards^a^				1.33 (0.92–1.94)
Model fit AIC, p-value^c^	212.6, 0.002	205.1, 0.002	204.5, 0.106	206.8, 0.219

^a^higher scores are favourable

^b^lower scores are favourable

^c^Model Akaike’s Information Criterion (AIC) and p-value of likelihood ratio test comparing Model fit with the less complex nested Model, i.e. Model 1 compared with a crude Model, Model 2 compared with Model 1, Model 3 compared with Model 2, and Model 4 compared with Model 3

Sensitivity analyses showed that the use of ever binge drinking as the outcome did not change the conclusions, as odds ratios for low compared with high educational track did not decrease between models (OR model 1 = 5.34, 95% CI = 1.97–14.47; OR model 4 = 5.62, 95%CI = 1.45–21.83). Results for weekly smoking were also similar to those of more than monthly smoking (OR model 1 = 6.69, 95% CI = 2.52–17.76; OR model 4 = 3.65, 95% CI = 1.13–11.79). The relationship between educational track and ever smoking was weaker and not statistically significant (OR model 1 = 1.89, 95%CI = 0.90–4.01; OR model 4 = 1.19, 95% CI = 0.46–3.13).

## Discussion

### Key findings

Smoking and binge drinking were more prevalent in students in the low educational track compared to those in the high educational track. The association between educational track and binge drinking was slightly reduced after adjustment for self-control and cognitive ability, but remained unchanged with all the cognitive functioning variables included. The association between educational track and smoking was reduced to a greater extent after adjustment for self-control and cognitive functioning, especially cognitive ability. However, inequalities in smoking remained substantial.

### Evaluation of potential data problems

The study used a convenience sample of eight schools. The study population may therefore not be representative for the general Dutch adolescent population. For example, ethnic minority groups were underrepresented. However, we included schools from cities and towns from different parts of the country, that had a mean income that was comparable with the Netherlands as a whole. Also, the three main educational tracks in the Dutch educational system were all included. Studies with larger and more diverse samples are needed to assess the generalizability of our findings for the Netherlands.

The cross-sectional study design does not provide information to assess the causality of the observed associations. As self-control and cognitive ability develop in early childhood and substance use is initiated later in life [34, 35], it is likely that self-control and cognitive ability predict substance use. A longitudinal study from New-Zealand found that childhood self-control predicts substance dependence in adulthood [36]. However, binge drinking may affect the cognitive performance of young people [37] and in a sample of adult smokers, smoking seemed to enhance self-control [38]. Moreover, a twin study in Finnish adults showed that smoking is negatively related to long-term labour market outcomes [39]. Reverse causation or bi-directionality of associations can therefore not be ruled out. Moreover, we cannot rule out that drinking and smoking are only indirectly related to self-control and cognitive functioning, due to common underlying factors related to these concepts, such as parenting practices or personality traits that were not measured in the data.

Smoking and binge drinking were self-reported. Reporting of socially desirable answers may lead to an underestimation of the prevalence of smoking and drinking [40]. However, in our study, students were assured that the survey was anonymous and that neither teachers nor parents would be informed of individual answers. Moreover, the dichotomous classifications of substance use that we applied in this paper are less vulnerable to reporting errors than more detailed classifications. Likewise, self-control was measured via a self-report survey and may therefore be subject to reporting bias. However, a in a meta-analysis of 236 studies, self-report questionnaires for self-control had a higher internal validity than delay of gratification tasks and executive function tasks [41].

### Comparison to previous studies and interpretation of results

We found that self-control was inversely associated with smoking and binge drinking, also after controlling for socio-demographic variables and cognitive functioning. Previous studies also found an inverse relationship between self-control and smoking and binge drinking [23–25]. For example, a longitudinal study by King et al. found that adolescents who had developed self-control problems over time were more likely to report smoking and binge drinking [25].

Cognitive ability was associated with smoking and educational track, and therefore weakened the association between educational track and smoking. However, this was not found for binge drinking. This might be due to the difference in the health beliefs of alcohol use and smoking in Western society [42]. While tobacco smoking is nowadays viewed by most adolescents as a health hazard and irresponsible behaviour, alcohol use is considered by many as a normal part of social life [42, 43]. Adolescents with higher intelligence or in high educational tracks may therefore be more inclined to protect their health against smoking than against alcohol use [43].

In our data, reaction time and memory span did not play a significant role in educational inequalities in smoking and binge drinking. These measurements for cognitive functioning have rarely been used in alcohol and tobacco research and their association with substance use may be converse to what we expected. Binge drinking during adolescence has a negative influence on memory span tests among females, but a positive influence among males [37]. Moreover, young adults who were heavy drinkers had faster reaction times than non-heavy drinkers [44]. Further studies should elucidate the nature of these unexpected relationships.

Self-control and cognitive functioning did not play a role in differences between educational tracks in binge drinking, and played a minor role in differences in smoking. Various other explanations have been developed for educational differences in substance use. Differences may originate from variations in social context and socialization processes, with those in lower tracks experiencing greater peer pressure to start using alcohol, tobacco, and other substances [45, 46]. High prevalence rates of smoking and binge drinking within the lower educational track may sustain itself because of its direct effect on social norms and peer pressure, resulting in freshmen adopting the view that drinking and smoking are normal behaviours.

School environment may also be an important factor to take into consideration. Stronger school policies may be associated with lower adolescent smoking rates [47] and lower adolescent binge drinking rates, especially among adolescents with a lower school attachment [48]. Moreover, parental substance use and parenting style may also influence their offspring’s substance use [49, 50]. Parenting styles characterized by warmth, care and positive emotional attachment lower the odds of adolescent substance use [50] and may be more common in some educational groups than others [51, 52].

Some authors argue that educational differences in smoking and binge drinking are entrenched in inequalities in society at large. These inequalities already start by differentiating young people into educational tracks at an early age [53, 54]. According to Elstad [15] the unpromising social prospects of those in lower tracks may lead to higher drinking and smoking prevalence due to “more need for stress-alleviating behaviours, less interest in the future … attempts to compensate lack of recognition in school by excelling in alternative social fields, and deliberate opposition to social authorities because of the experience of being rejected by them”.

## Conclusion

In this study population, pre-existing variations between adolescents in terms of self-control and cognitive functioning played a minor role in educational inequalities in smoking, but not in binge drinking. Even though this study provides some empirical support for indirect selection mechanisms [15], the evidence is still limited. To a large extent, inequalities in smoking and binge drinking in this adolescent population appear to be caused by factors that result from adolescents’ experience of being enrolled in a low educational track, including peer influences, a less supportive school environment, and less promising social prospects.
